# Left atrial dissection after a supra-annular mitral valve replacement for endocarditis

**DOI:** 10.1186/s13019-022-02097-x

**Published:** 2023-02-15

**Authors:** Valentin de Villiers de la Noue, Guillaume Théry, Laurent Faroux, Nasreddine Belkessa, Sylvain Rubin, Bruno Mourvillier, Antoine Goury

**Affiliations:** 1 Intensive Care Medicine, Reims University Hospital, Reims, France; 2 Department of Cardiology, Reims University Hospital, Reims, France; 3 Department of Radiology, Reims University Hospital, Reims, France; 4 Thoracic and Cardiovascular Surgery, Reims University Hospital, Reims, France

**Keywords:** Left atrial dissection, Transoesophageal echocardiography, Cardiac CT-scan

## Abstract

**Background:**

Left atrial dissection is a rare and a potentially fatal complication of cardiac surgery. Multi-modal imagery is helpful for the diagnosis and to guide the treatment.

**Case presentation:**

We report the case of a 66-year-old female patient who underwent a combined mitral and aortic valve replacement for degenerative valvular disease. She presented an infectious endocarditis revealed by a third-degree atrioventricular bloc and had a redo mitral- and aortic valve replacement. Mitral valve was inserted in supra-annular position due to annular destruction. Post-operative course was marked by a refractory acute heart failure explained by a left atrial wall dissection confirmed by transoesophageal echocardiography and synchronized cardiac CT-scan. Surgical treatment was theoretically indicated but considering the high risk of a third surgery, a palliative care support was collegially decided.

**Conclusions:**

Left atrial dissection can occur after a redo surgery and supra-annular mitral valve implantation. Multi-modal imagery including transoesophageal echocardiography and cardiac CT-scan is helpful for the diagnosis.

## Introduction

Left atrial dissection (LAD) is a rare but potentially fatal complication of cardiac surgery, defined by a forced separation of the left atrial (LA) wall layers. Few cases are described after cardiac surgery and endocarditis [1]. In very rare cases complicating valve mitral replacement, LAD can be confounded or associated with left ventricle rupture.

LAD occurring after cardiac surgery are mainly described after mitral valve replacement or a combined aortic and mitral valve replacement [2]. The incidence of LAD after mitral valve surgery has been reported between 0.16 and 0.84% [3, 4]. In some cases, the mitral annulus can be damaged after debridement of calcified valves, inadvertent incision during the surgical procedure, oversizing or intense traction of the prosthesis on the annulus.

In case of a supra-annular insertion of the prosthetic valve, modifications of the shear forces or the unusual traction can lead to a LAD after wall dissection, a false aneurysm (FA) development, and sometimes a pulmonary vein obstruction causing heart failure and low-cardiac output [5].

LAD can be revealed by different symptoms such as dyspnoea, chest pain, palpitations, syncope, or even cardiac arrest. One of the most common presentations is a postoperative hemodynamic or acute respiratory failure.

## Case presentation

We report the case of a 66-year-old female patient suffering from acute heart failure due to severe aortic and mitral stenosis. She underwent a combined mitral and aortic valve replacement with bioprosthetic valves (respectively 29-mm Epic St Jude Medical and 21-mm Trifecta GT St Jude Medical) on October 7th, 2021 and was discharged after a 10-day hospitalization.

4 weeks later, she was admitted to hospital for asthenia and fever. A paroxystic third-degree atrioventricular block was diagnosed on the electrocardiogram and blood cultures were positive to *Enterococcus faecalis.* Transthoracic echocardiography (TTE) confirmed the presence of a double supracentimetric endocarditis on the two prosthetic valves with an aortic root abscess. CT-scan did not find any sign of septic embolism. She received an antibiotherapy with Ceftriaxone and Amoxicillin.

The patient underwent a replacement of the aortic and mitral prosthetic valves and the implantation of a leadless pacemaker on November, 07th 2021.

Surgical examination confirmed the diagnosis of large vegetations of the two prosthetic valves and a massive destruction of the mitral annulus. The two prosthetic valves were replaced. A 21-mm Edwards Magna Ease valve was placed in aortic position. Nevertheless, because of the extensive deterioration of the mitral annulus, the 31-mm Edwards Magna Ease valve was inserted in supra-annular mitral position. Immediate post-operative transoesophageal echocardiography (TOE) was satisfying, with a good function of the new prosthetic valves. She was weaned from mechanical ventilation the next day.

The postoperative course was marked by an acute kidney injury needing continuous renal replacement therapy. One week after the surgery, she developed a severe hypoxemic respiratory failure due to a pulmonary oedema needing mechanical ventilation.

**Fig. 1:**
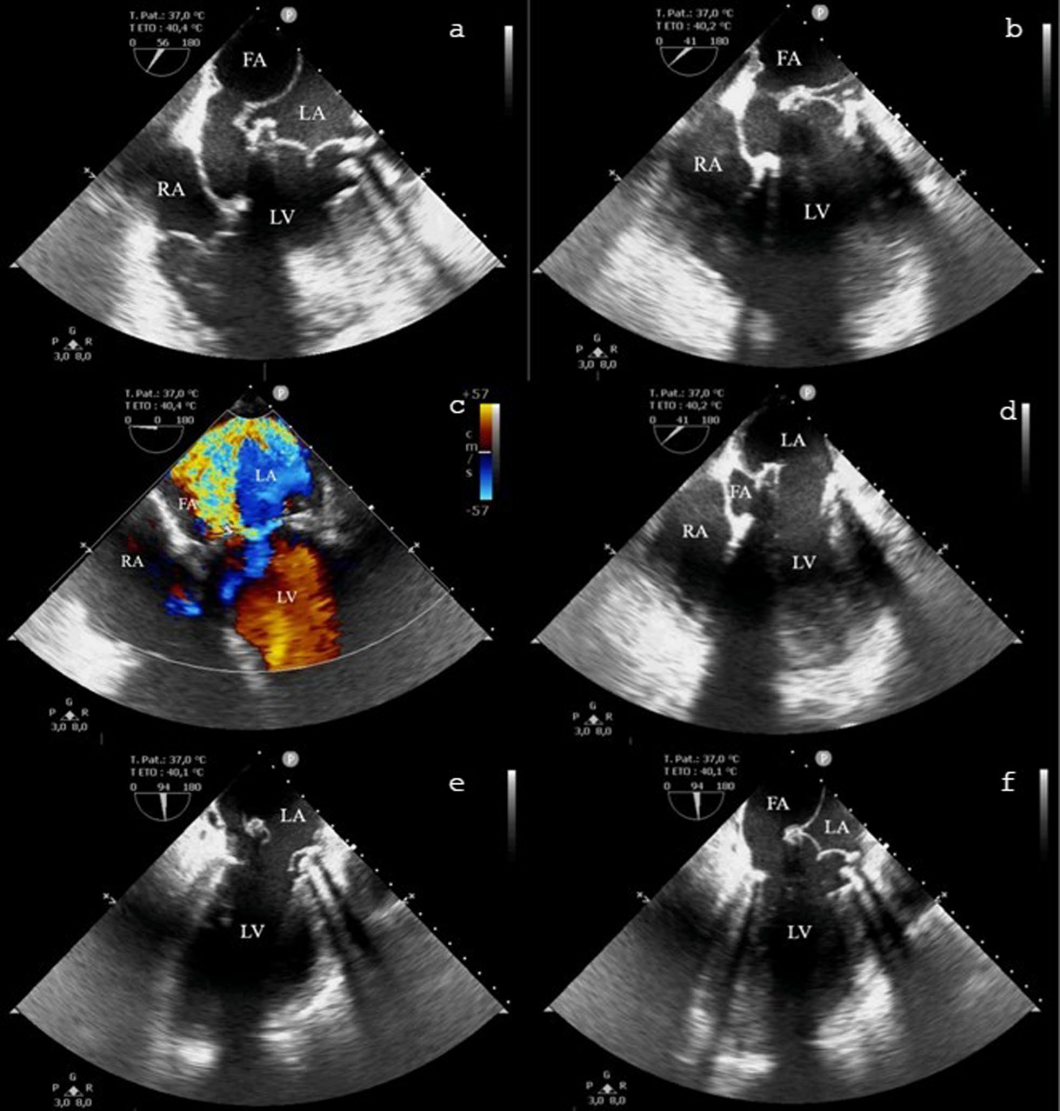
Left atrial dissection visualized in transesophageal echocardiography. **a**, **b**, **d** Mid-oesophageal 4-chamber view, the false aneurysm is squeezing the left atrium in systole (**a**, **b**), and drained in the left ventricle in diastole (**d**). **c** Mid-oesophageal 4-chamber view with colour-doppler illustrating the para-valvular leakage. **e**, **f** mid-oesophageal 4-chamber view in systole (**f**) and diastole (**e**). FA: false aneurysm, LA: left atrium, LV: left ventricle, RA: right atrium

**Fig. 2:**
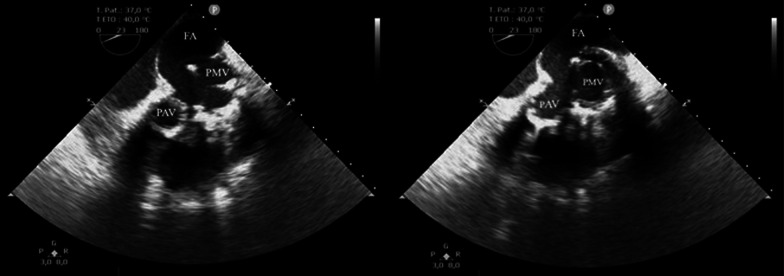
Transgastric transoesophageal echocardiography views; the false aneurysm is developed behind the prosthetic mitral valve and the left atrium. FA: false aneurysm, PAV: prosthetic aortic valve, PMV: prosthetic mitral valve

The TOE revealed a LA wall dissection and the creation of a new atrium cavity interpreted as a FA behind the LA posterior wall, squeezing the left atrium at every systole (Figs. 1, 2). The two new prosthetic valves were functional and unscathed without intra- nor para-valvular regurgitation. A severe left-ventricular regurgitant jet filled the FA pushing the LA posterior wall through the LA, probably causing pulmonary veins obstruction (Fig. 1). No shunts were detected by the bubble test and the aortic wall and the interatrial septum were normal.

**Fig. 3:**
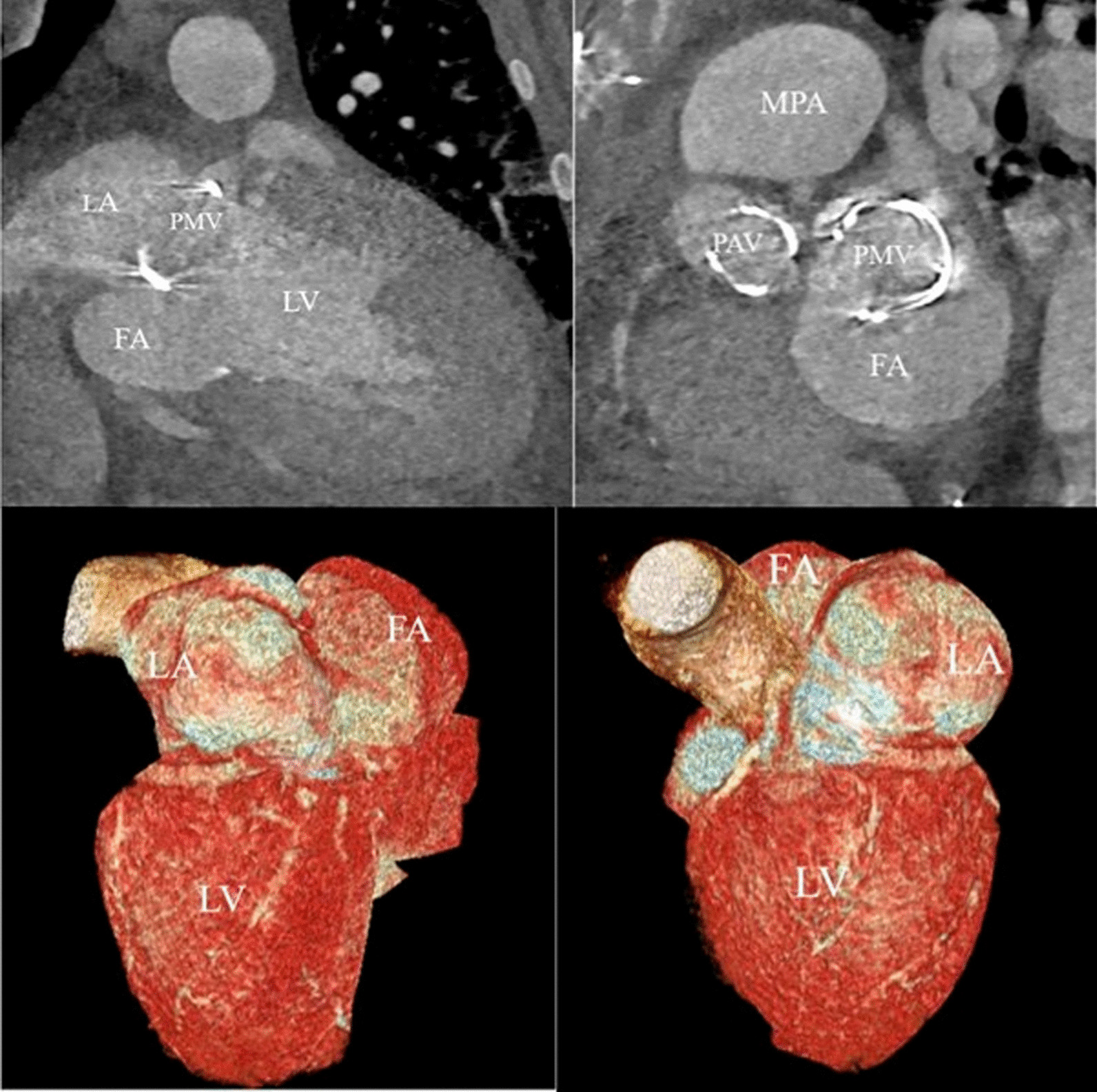
Cardiac CT-scan and 3D-reconstruction. The false aneurysm measures 65 × 40 × 30 mm, behind the prosthetic mitral valve and the left atrium. FA: false aneurysm, LA: left atrium, LV: left ventricle, PAV: prosthetic aortic valve, PMV: prosthetic mitral valve

Contrast-enhanced synchronized cardiac CT-scan (128-slice) was realized to perform a 3D reconstruction imaging to guide a potential surgery. It confirmed a FA measuring 65 × 40 x 30 mm behind the LA with a mass effect on it, communicating with the left ventricle outflow tract through a 23-mm defect, close to the mitral prosthetic valve (Fig. 3). Pulmonary veins were correctly inserted in the right atrium.

Unfortunately, weaning from mechanical ventilation was unsuccessful. Considering the high risk of a third surgery, the impossibility of a percutaneous treatment facing a very large collar and false aneurysm and the worsening of the clinical course, a palliative care support was collegially decided and the patient finally died.

## Discussion

LAD is a rare complication of valve surgery. This case reported a LAD occurring after a combined mitral and aortic valvular replacement with the emergence of a large cavity in the LA. Here, the initial predisposed factor was the mitral annular destruction needing a supra-annular implantation of the prosthetic valve in the weak portion of the atrium wall. Consequently, the prosthetic valve caused overtension on the atrium wall and the increase of shear force at every systole. Using a pericardial patch to repair the annulus prior to the implantation of the new prosthesis could have been a way to avoid LAD instead of inserting but this solution was not retained during the 2nd surgery.

No guidelines exist about how to treat LAD. The final decision was based on individual basis. Medical or surgical supports could be chosen regarding symptoms and especially hemodynamic situation. In most cases, urgent surgery was undertaken (73.4%) [4]. In other cases, scheduled surgery was performed for patients with delayed or late presentations. Surprisingly, the mortality rate after repair surgery is low (12.7%) according to this type of complication. Finally, for the quarter of the patients (24.1%) which did not experience symptoms or hemodynamic instability, a conservative treatment was decided with favourable outcomes.

In order to avoid shear force on the atrial wall and in consequence LAD, an annular reconstruction with an autologous pericardial patch can be made [6].

New ways of treatment could be discussed with progress of minimally invasive approaches in the manner of percutaneous paravalvular leak closure [7]. To the best of our knowledge, no cases had been reported, but facing a paravalvular leakage in supra-annular position with a very large entry, percutaneous approach seemed not to be an appropriate treatment.

TOE is the gold standard to provide morphologic and hemodynamic data. Nevertheless, Cardiac CT-scan and 3D-reconstruction can be a useful tool to understand heart anatomy and to guide surgical treatment in visualizing the geometric relationships to plan and execute complex surgical procedures via minimally invasive or standard approaches. Cardiac CT-scan is a reliable technique for assessing risk and planning transcatheter valve interventions for mitral and aortic valves [8].

However, even though imagery explorations helped for the diagnosis, the patient did not undergo curative treatment of LAD because a high risk surgery in context of multiple organ failure and in accordance with family choices. Post-mortem anatomic analysis could have clarified the exact pathology of the atrium but had not been realized.

## Conclusion

LA dissection is a rare but potentially fatal complication after cardiac valve replacement [1]. Refractory acute heart failure is one of the clinical patterns. Echocardiography (TTE and TOE) and Cardiac CT-scan with 3D reconstruction are key tools to make a prompt diagnosis and discuss the best management to avoid worsening course and fatal outcomes.

## Data Availability

All data generated or analysed during this study are included in this published article.
